# Establishment and Validation of a Model of Indwelling Nasogastric Tube in Patients After Acute Ischemic Stroke

**DOI:** 10.1002/brb3.71874

**Published:** 2026-11-05

**Authors:** Yaying Zeng, Wenbo Zhang, Yudi Han, Yijia Ye, Peiliang Zhang, Wujie Shi, Jianbin Weng, Zhipeng Shen, Zhi‐Lin Chen

**Affiliations:** ^1^ Department of Psychiatry The First Affiliated Hospital of Zhejiang Chinese Medical University (Zhejiang Provincial Hospital of Traditional Chinese Medicine) Hangzhou China; ^2^ Department of Neurosurgery The Children's Hospital of Zhejiang University School of Medicine, National Clinical Research Center For Child Health Hangzhou China; ^3^ Department of Neurology Translational Research Institute of Brain and Brain‐Like Intelligence, Shanghai Fourth People's Hospital, School of Medicine Tongji University Shanghai China; ^4^ Department of Neurology, Shanghai Tongji Hospital, School of Medicine Tongji University Shanghai China; ^5^ State Key Laboratory of Neurology and Oncology Drug Development Nanjing China

**Keywords:** acute ischemic stroke, dysphagia, machine learning, nasogastric tube, prediction model

## Abstract

**Background:**

Dysphagia is common after stroke, and accurately predicting its duration is essential for guiding therapeutic decisions. Timely indwelling nasogastric tube (NGT) placement is critical for patient prognosis. The purpose of this study was to develop and validate a predictive model for indwelling NGT requirements in patients with acute ischemic stroke (AIS).

**Materials and methods:**

From 2020 to 2022, we retrospectively analyzed the clinical data of 553 patients with AIS. The least absolute shrinkage and selection operator (LASSO) regression and multivariate logistic regression were employed to identify predictors. Multiple machine learning (ML) classifiers were integrated to identify the optimal model, followed by validation. Finally, Shapley Additive exPlanations (SHAP) was applied for model interpretation and personalized risk assessment.

**Results:**

A total of five variables—patient's National Institutes of Health Stroke Scale (NIHSS) score, age, free thyroxine (fT4), neutrophil‐to‐lymphocyte ratio (NLR), and stroke location, were derived from Least Absolute Shrinkage and Selection Operator (LASSO) regression and logistic regression as independent predictors associated with indwelling NGT requirement in AIS patients. Among nine machine learning classifiers, random forest demonstrated optimal performance. The model achieved an AUC of 0.850 in fivefold cross‐validation within the training cohort and 0.853 in the independent testing cohort.

**Conclusion:**

The proposed model serves as a user‐friendly prognostic tool for reliably predicting swallowing dysfunction while offering interpretable results. It may assist clinicians in making NGT placement decisions for AIS patients, facilitating personalized care.

## Abstract 2

We developed and validated an interpretable machine‐learning model to predict indwelling nasogastric tube (NGT) requirements in acute ischemic stroke (AIS). LASSO and logistic regression identified five predictors (NIHSS score, age, fT4, NLR, stroke location); among nine classifiers, Random Forest performed best (AUC 0.850 training, 0.853 testing). SHAP‐based personalized risk assessment supports timely NGT placement decisions.

## 1 Introduction

Stroke represents a leading cause of severe disability worldwide, with an annual incidence exceeding 13 million cases (Foley et al. 2008). Poststroke dysphagia (PSD) is a prevalent complication, and elucidating its recovery trajectory is essential for prognostic evaluation and care planning. PSD can lead to increased risk of dehydration, malnutrition, and stroke‐associated pneumonia (Galovic et al. 2019; Dziewas et al. 2021). Furthermore, PSD contributes to prolonged hospital stays, elevated healthcare costs, and increased mortality (Bonilha et al. 2026). Malnutrition secondary to PSD may compromise immune function, wound healing capacity, muscular strength, and psychological well‐being. Consequently, ensuring adequate nutritional support for all affected patients represents a critical clinical priority.

Given the significant clinical implications of PSD, a thorough understanding of its natural history and recovery patterns is imperative. While oral nutritional supplementation suffices for some patients, others necessitate enteral feeding via nasogastric tube (NGT). Guidelines recommend the use of NGT for feeding when resumption of oral feeding is unlikely within 1 week (Stroud et al. 2003). However, NGT placement does not benefit all stroke patients equally. NGT retention frequently causes nasopharyngeal discomfort, often accompanied by oral pain, thirst, dysphagia, and vocal cord dysfunction. The localized pressure effects may induce sinusitis, otitis media, nasal erosions, or abscess formation. Short‐term esophageal complications encompass reflux‐induced esophagitis, abrasions, and ulcers, whereas long‐term sequelae may include severe strictures (Alverdy et al. 2026). Therefore, clinicians need to predict the duration of dysphagia in order to place NGT at the appropriate time. Nutritional support should be given as early as possible (within 72 h of stroke), with a focus on early and accurate prediction of the patient's need for NGT at 1 week. Current NGT decision‐making demonstrates considerable variability, frequently relying on clinician subjectivity.

Machine learning (ML) has emerged as a transformative approach in medicine, with growing applications in precision medicine and diagnostic support systems (Greener et al. 2022; Handelman et al. 2018). This study aims to enhance predictive accuracy through advanced ML algorithms, thereby improving prognostic stratification, guiding therapeutic decisions, and facilitating personalized patient management.

## 2 Materials and Methods

### 2.1 Study Population

We conducted a retrospective cohort study analyzing clinical data from 553 acute ischemic stroke (AIS) patients admitted to Shanghai Tongji Hospital between January 2020 and December 2022. The study was approved by the Ethics Committee of the Shanghai Tongji Hospital and complied with the Helsinki Declaration. Only patients who do not require neurosurgical intervention meet the inclusion criteria. This study excluded patients with preexisting dysphagia or any accompanying diseases that could lead to dysphagia, and all patients underwent swallowing function assessment within 48 h of admission. Exclusion criteria: (1) Patients lost to follow‐up; (2) Patients with chronic pneumonia, active infections, or fever within 2 weeks prior to admission; (3) History of hematologic diseases, severe liver disease, thyroid disease, tumors, or immunosuppressive therapy; and (4) Patients with significant loss of consciousness after a stroke.

### 2.2 Dysphagia Assessment

*Dysphagia assessment and follow‐up protocol*: Swallowing function was assessed using the Functional Oral Intake Scale (FOIS), a validated 7‐point scale (1 = *no oral intake*, 7 = *full oral diet without restrictions*). Severe dysphagia was operationally defined as FOIS level ≤ 4 (representing tube‐dependent or partial oral intake), with these patients requiring continued NGT feeding support. The primary endpoint was persistent severe transoral dysphagia (FOIS score ≤ 4) at 1‐week follow‐up, based on the guideline that if transoral dysphagia persisted for 1 week, initiation of NGT feeding was recommended (Kirby et al. 1995).

*Follow‐up process and timing*: All patients underwent baseline FOIS assessment by a licensed rehabilitation physician within 48 h of admission during the initial clinical swallowing screening. Follow‐up assessment was performed during Days 5–7 post‐admission during routine morning ward rounds. The timing was standardized to minimize diurnal variation in patient alertness and cooperation. The rehabilitation physician evaluated the patient's current oral intake status, documented the FOIS level in the electronic medical record, and made a clinical recommendation regarding NGT retention or removal. NGT placement or removal decisions were made by the attending neurologist in consultation with the rehabilitation team.

*Evaluator qualifications and standardization*: FOIS assessments were performed by rehabilitation physicians who had completed standardized departmental training in dysphagia evaluation, including didactic instruction on FOIS scoring criteria and supervised bedside practice. All evaluating physicians held a practicing license and had a minimum of 2 years of clinical experience in stroke rehabilitation. The same rehabilitation physician typically performed both baseline and follow‐up assessments for each patient to ensure continuity.

*Blinding*: Given the retrospective design and the integration of FOIS assessment into routine clinical care, evaluators were not blinded to patient clinical characteristics or treatment during FOIS scoring. To minimize potential observer bias, a standardized FOIS scoring protocol with explicit operational definitions for each level was uniformly applied. For neuroimaging interpretation (CT/MRI), two neurologists independently reviewed all scans blinded to clinical data, FOIS scores, and NGT status, with discrepancies resolved by consensus. For data extraction and statistical analysis, the analyst was blinded to clinical outcomes during the initial coding phase.

### 2.3 Development of the Statistical Model

All patients underwent laboratory examinations on an empty stomach the next morning and completed CT examinations within 24 h of admission. Stroke localization and lesion burden determined by MRI prior to discharge. Cerebrovascular events were classified according to the Trial of Org 10,172 in Acute Stroke Treatment (TOAST) criteria (D'Netto et al. 2023). For analytical purposes, the TOAST subtypes were dichotomized into large‐artery atherosclerosis (LAA) and non‐LAA categories, with LAA representing the predominant subtype. Stroke location was categorized into supratentorial and infratentorial types. Potential characteristics of the disease were selected, including baseline data on the patient: age, sex, National Institutes of Health Stroke Scale (NIHSS) score, body mass index (BMI), systolic blood pressure, diastolic blood pressure, history of hypertension, history of diabetes, history of coronary heart disease, history of hyperlipidemia, history of stroke, history of smoking, history of drinking, aspiration pneumonia, TOAST classification, and stroke location and number of focus, as well as laboratory tests: uric acid, vitamin D, total bilirubin, alanine aminotransferase, aspartate aminotransferase, creatinine, urea nitrogen, HbA1c, total cholesterol, triglycerides, low density lipoprotein, high density lipoprotein, C‐reactive protein, homocysteine, thyroid stimulating hormone, free triiodothyronine (fT3), free thyroxine (fT4), red blood cell, white blood cell, lymphocyte neutrophil‐to‐lymphocyte ratio (NLR), hemoglobin, platelet, and folic acid. Then, Least Absolute Shrinkage and Selection Operator (LASSO) and logistic regression are used to select NGT features.

In order to obtain the best prediction performance, nine models were constructed, including the extreme gradient boosting (XGBOOST), logistic regression, random forest (RF), AdaBoost, light gradient boosting machine (LightGBM), complementary neural networks (CNB), decision trees, support vector machines (SVM), and the k‐nearest neighbor machine (KNN). Continuous variables with normal distribution were presented as mean ± standard deviation (SD), compared using independent samples *t*‐tests. Non‐normally distributed variables were analyzed using the Mann–Whitney *U* test. Categorical data were summarized as counts (percentages) and compared using Pearson's *χ* ^2^ test. Finally, the most important features were filtered by LASSO regression analysis and logistic regression, and nine models were built based on their feature sets.

Python (sklearn 0.23) was used to construct the area under the receiver operating characteristic (ROC) curve, and R software (rmda 1.6) was used to plot the decision curve analysis (DCA). We used Python (sklearn 0.23) to build calibration curves, measure the predictive power of the models, and perform a comprehensive evaluation of the predictive model. Plotting precision recall (PR) curves using Python (sklearn 0.23) for evaluating model performance and providing a valuable addition to existing model evaluation methods.

### 2.4 Training, Validation, and Testing of Optimal Models

The 553 patients from a single center were randomly divided into a training cohort (442 patients, 80%) and an independent testing cohort (111 patients, 20%) using a stratified randomization approach to preserve the distribution of the outcome variable. All data were derived from a single center, no external validation cohort was available. Within the training cohort, a fivefold cross‐validation strategy was employed for model selection and hyperparameter tuning. In each fold, the training cohort was further partitioned into five subsets, four subsets were used for training and the remaining subset served as an internal validation fold. This process was repeated five times to ensure that each subset was used for validation once. Model performance was averaged across the five folds to obtain stable estimates. After the optimal model was selected based on cross‐validation performance, the final model was evaluated on the held‐out testing cohort to assess its generalizability. ROC curves were plotted to assess model discrimination for the training cohort (cross‐validation performance) and the independent testing cohort. SHAP interpretations of model significance and contribution were plotted using Python (shap 0.47.0), and model results were interpreted by calculating the contribution of each feature to the predicted results. Furthermore, individual‐level SHAP analysis was performed to demonstrate feature contributions at the case level, complementing the aggregate model performance metrics.

## 3 Results

A total of 42 variables were included, all of which were not statistically different from each other (Table 1). LASSO regression analysis of all independent variables with the presence or absence of NGT as the dependent variable was performed on the training cohort (Figure 1). The results identified 14 independent variables with non‐zero coefficients: BMI, NIHSS score, age, low density lipoprotein, aspartate aminotransferase, total bilirubin, fT4, fT3, NLR, history of diabetes, history of hyperlipidemia, aspiration pneumonia, TOAST classification, and stroke location. Multivariate logistic regression analyses were performed on the 14 independent variables mentioned above. Finally, only NIHSS score, age, fT4, NLR, and stroke location were identified as independent predictors significantly associated with NGT requirement (*p* < 0.05), as detailed in Table 2.

**TABLE 1:** Clinical baseline characteristics of the training cohort and testing cohort.

Variable	Training cohort (*n* = 442)	Testing cohort (*n* = 111)	*p*
Sex, *n* (%)			
Male	65.2	64.9	0.954
Female	34.8	35.1	
Hypertension, *n* (%)			
No	30.8	26.1	0.339
Yes	69.2	73.9	
Diabetes, *n* (%)			
No	77.6	73.9	0.405
Yes	22.4	19.3	
Coronary heart disease, *n* (%)			
No	93.2	96.4	0.212
Yes	6.8	3.6	
Hyperlipidemia, *n* (%)			
No	94.8	92.8	0.412
Yes	5.2	7.2	
Stroke history, *n* (%)			
No	90.0	90.1	0.989
Yes	10.0	9.9	
Toast, *n* (%)			
LAA	52.0	57.7	0.289
Non‐LAA	48.0	21.8	
Stroke location, *n* (%)			
Supratentorial	79.7	79.3	0.819
Subtentorial	21.7	20.3	
Number of focus, *n* (%)			
Single	181 (45.363)	27 (36.986)	0.185
Multiple	218 (54.637)	46 (63.014)	
Smoke, *n* (%)			
No	50.2	49.5	0.899
Yes	49.8	50.5	
Drink, *n* (%)			
No	54.5	52.3	0.668
Yes	45.5	47.7	
Pneumonia, *n* (%)			
No	88.0	90.1	0.540
Yes	12.0	9.9	
Uric acid, *n* (%)			
246.0–326.0	147 (33.485)	28 (27.451)	0.061
> 326.0	152 (34.624)	28 (27.451)	
≤ 246.0	140 (31.891)	46 (45.098)	
Age, mean (±SD)	60.24 ± 10.54	59.82 ± 10.47	0.507
Body mass index, mean (±SD)	24.19 ± 3.34	24.45 ± 2.94	0.296
Systolic blood pressure, mean (±SD)	155.15 ± 22.88	158.06 ± 21.32	0.132
Diastolic blood pressure, mean (±SD)	83.28 ± 12.73	84.51 ± 13.64	0.316
VitD, median, mean (±SD)	49.47 ± 25.32	45.84 ± 24.96	0.182
Total bilirubin, mean (±SD)	13.24 ± 8.52	12.93 ± 6.15	0.804
Alanine aminotransferase, mean (±SD)	20.71 ± 11.71	22.45 ± 13.71	0.332
Aspartate aminotransferase, mean (±SD)	22.96 ± 9.24	24.71 ± 14.84	0.509
Urea nitrogen, mean (±SD)	5.08 ± 1.45	5.42 ± 1.71	0.093
Creatinine, mean (±SD)	72.58 ± 19.33	77.17 ± 23.35	0.151
HbA1c, mean (±SD)	6.43 ± 1.62	6.42 ± 1.44	0.559
Total cholesterol, mean (±SD)	4.79 ± 1.11	4.83 ± 1.10	0.0.747
Triglycerides, mean (±SD)	1.66 ± 0.87	1.83 ± 1.09	0.116
High density lipoprotein, mean (±SD)	1.15 ± 0.28	1.09 ± 0.25	0.088
Low density lipoprotein, mean (±SD)	2.80 ± 0.90	2.86 ± 0.94	0.692
C‐reactive protein, mean (±SD)	7.13 ± 14.05	4.39 ± 7.03	0.620
Homocysteine, mean (±SD)	10.18 ± 5.67	9.90 ± 5.72	0.664
Thyroid stimulating hormone, mean (±SD)	1.84 ± 1.42	1.80 ± 1.29	0.790
fT4, mean (±SD)	9.25 ± 4.01	9.23 ± 4.15	0.857
fT3, mean (±SD)	3.89 ± 0.93	3.90 ± 0.98	0.816
Red blood cell, mean (±SD)	4.49 ± 0.52	4.59 ± 0.55	0.086
White blood cell, mean (±SD)	6.85 ± 1.95	6.84 ± 1.84	0.923
Neutrophils, mean (±SD)	0.63 ± 0.22	0.66 ± 0.36	0.441
Lymphocyte, mean (±SD)	0.28 ± 0.13	0.33 ± 0.30	0.473
Neutrophil‐to‐lymphocyte ratio, mean (±SD)	2.68 ± 1.77	2.56 ± 1.54	0.713
Hemoglobin, mean (±SD)	136.54 ± 16.19	139.25 ± 17.48	0.134
Platelet, mean (±SD)	207.41 ± 55.35	202.35 ± 51.12	0.375
NIHSS, mean (±SD)	3.98 ± 3.31	3.94 ± 3.14	0.992
Folic acid, mean (±SD)	7.66 ± 5.35	7.01 ± 4.21	0.453

**FIGURE 1:**
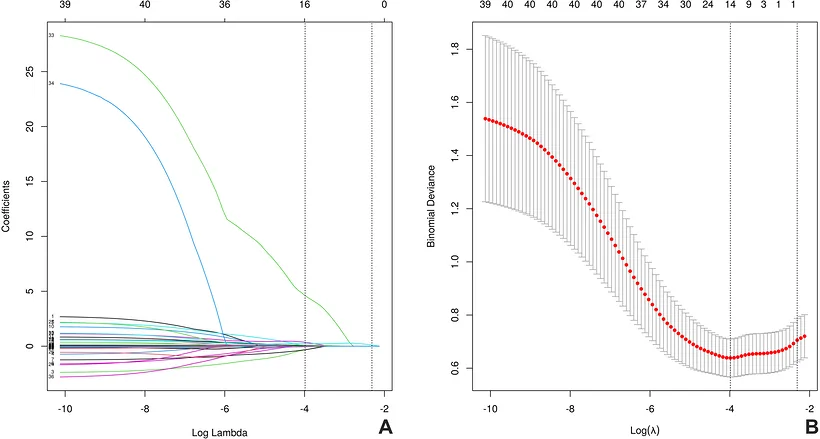
LASSO regression analysis was used to select feature factors.

**TABLE 2:** Multivariate logistic regression analysis after LASSO analysis.

Predictor	SE	*Z*	*p*	Odds ratio	95%CI
Body mass index	0.049	−0.364	0.716	0.982	0.892–1.081
NIHSS	0.043	4.483	< 0.010	1.215	1.116–1.325
Age	0.017	4.179	< 0.010	1.074	1.040–1.113
Low density lipoprotein	0.167	1.677	0.094	1.324	0.953–1.842
Aspartate aminotransferase	0.012	1.669	0.095	1.020	0.996–1.044
Total bilirubin	0.022	−0.039	0.969	0.999	0.955–1.038
fT4	0.048	2.199	0.028	1.111	1.014–1.226
fT3	0.199	1.893	0.058	1.457	0.963–2.148
Neutrophil‐to‐lymphocyte ratio	0.094	5.028	< 0.010	1.601	1.343–1.942
Diabetes	0.353	−0.054	0.957	0.981	0.483–1.940
Hyperlipidemia	0.597	0.378	0.705	1.254	0.358–3.822
Pneumonia	0.409	−0.040	0.968	0.984	0.428–2.149
TOAST	0.312	1.022	0.307	1.376	0.744–2.544
Stroke location	0.345	1.957	0.050	1.963	0.990–3.844

After identifying these five variables, ML models were used to predict poststroke NGT. We evaluated the predictive models using AUC, DCA curves, PR curves, and calibration curves. The evaluation of the nine models was shown in Figure 2. After comprehensive comparison, RF was selected as the best fitting model. The RF model achieved an in‐sample training AUC of 1.000 ± 0.000. DCA indicated that RF ranked second only to LightGBM, whereas the PR curve revealed marginally lower efficacy of RF compared to LightGBM. In calibration analysis, RF exhibited the smallest SD among all models, along with the highest validated AUC. Collectively, these results demonstrate that the RF model outperforms the other eight ML models in terms of generalization performance and calibration stability.

**FIGURE 2:**
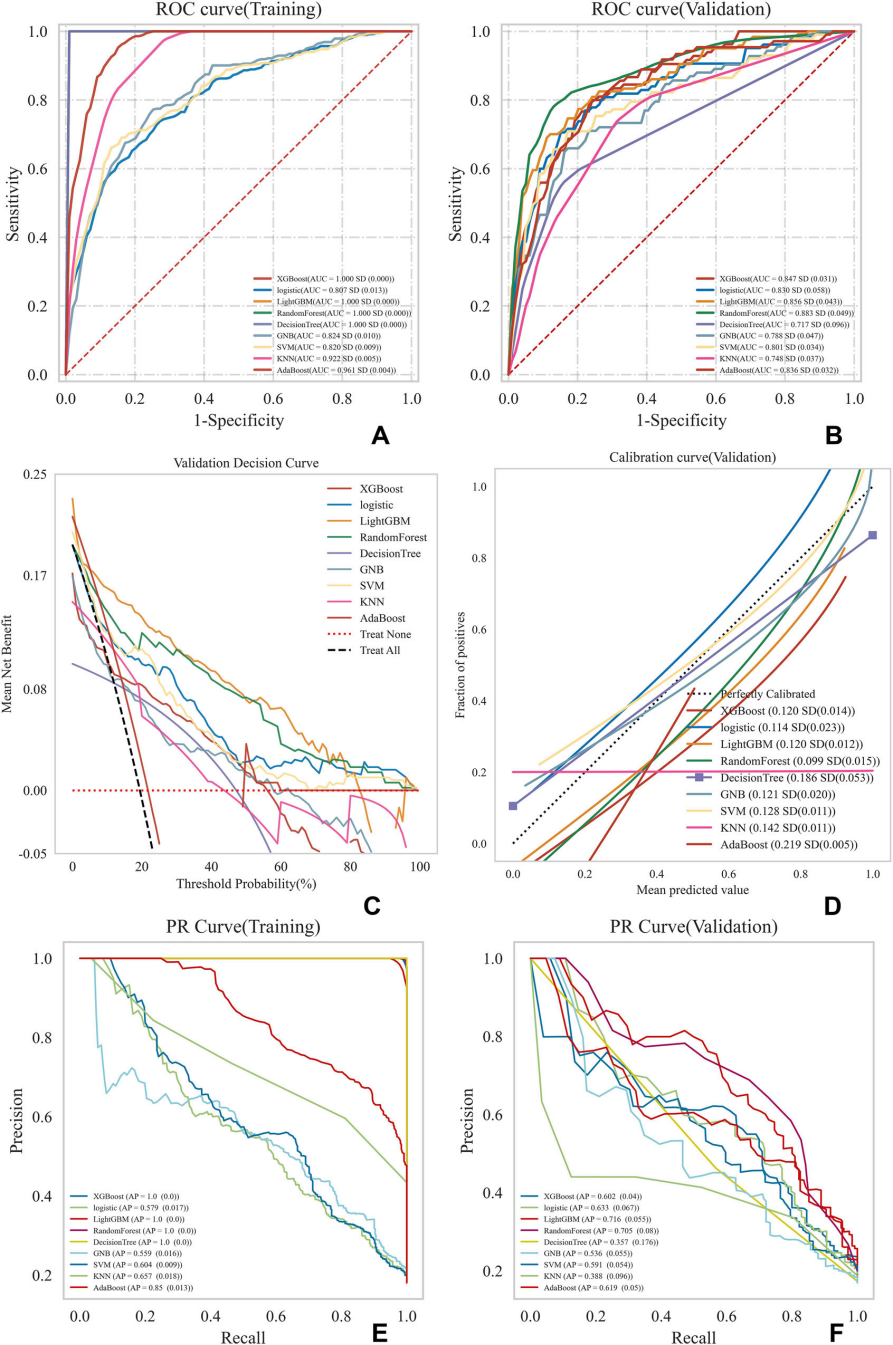
Comparison of results from nine machine learning models. (A) Training cohorts ROC and AUC and (B) testing cohorts ROC and AUC. (C, D) DCA and calibration curves for each model, respectively. (E) Training cohort PR curves and (F) testing cohort PR curves.

### 3.1 The Best Model Building and Evaluation

RF analysis and fivefold cross‐validation were performed on the training cohort, and the final model was evaluated on the independent testing cohort, as shown in Figure 3. The in‐sample training performance achieved a perfect AUC of 1.000 ± 0.000, which reflects the high capacity of the RF algorithm to fit training data and indicates some degree of overfitting. The mean fivefold cross‐validation AUC was 0.850 ± 0.041, and the independent testing cohort AUC was 0.853. The close agreement between cross‐validation performance and testing cohort performance (difference of 0.003) is the primary indicator of model generalizability. Although the training‐set AUC of 1.000 suggests that the model fully captured patterns within the training data, including potential noise, the consistency between internal cross‐validation and external testing performance demonstrates acceptable model stability and generalization capability for clinical application.

**FIGURE 3:**
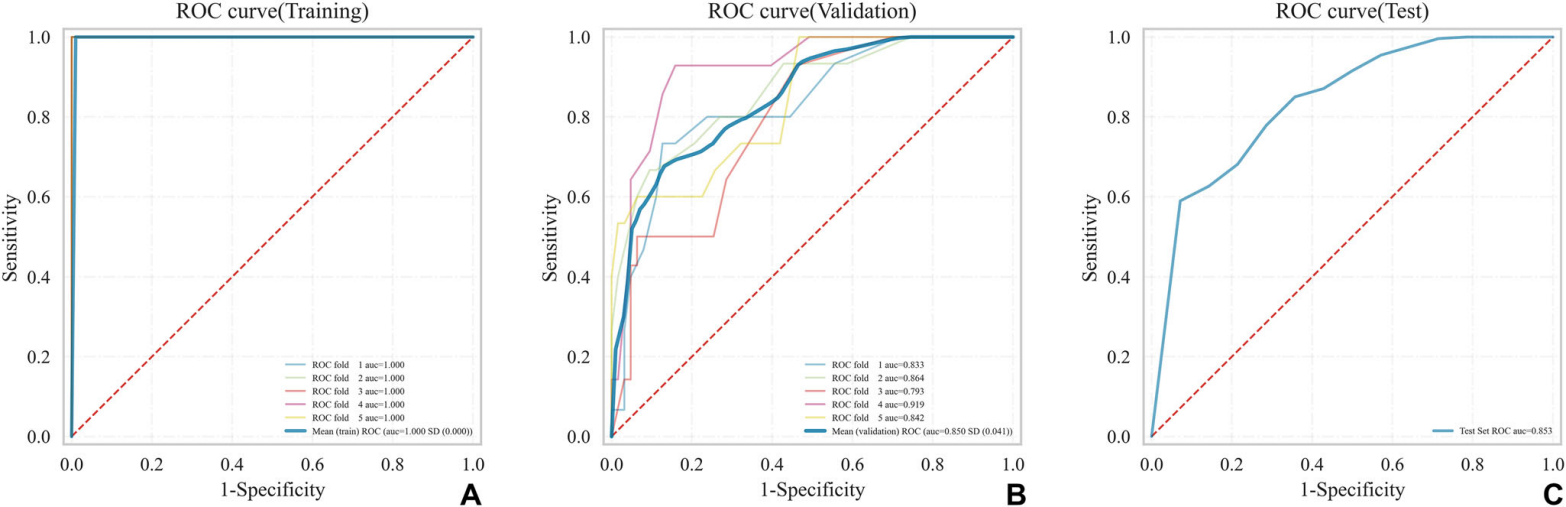
Random forest model training, validation, and testing. RF analysis and fivefold cross‐validation were performed on the training and validation cohort, and the blue line shows the average of the five sets of AUCs (A) Training cohort ROC curves. (B) Validation cohort ROC curves. (C) Testing cohort ROC curve.

### 3.2 The SHAP to Model Interpretation

To visually explain the selected variables, we use SHAP to illustrate how these factors predict NGT formation in the model. Figure 4A displays the five predictive variables included in our final model. In the SHAP summary plot (Figure 4A), each feature's importance is represented horizontally, with individual patient contributions depicted as colored dots (red: high‐risk values; blue: low‐risk values). Figure 4B presents the ranked importance of the five prognostic factors, where the *x*‐axis represents SHAP values as a measure of predictive contribution. The SHAP Force Plots were (Figure 4C,D) used to interpret individual predictions. Red bars indicate positive contributions and blue bars indicate negative contributions. The higher the contribution value, the longer the bar. The baseline values in the figure are the average output values of the model, and the predictions in the middle demonstrate the final results when the features are combined. These visualizations collectively demonstrate both the relative importance and directional effects (positive/negative) of each feature in the prediction model.

**FIGURE 4:**
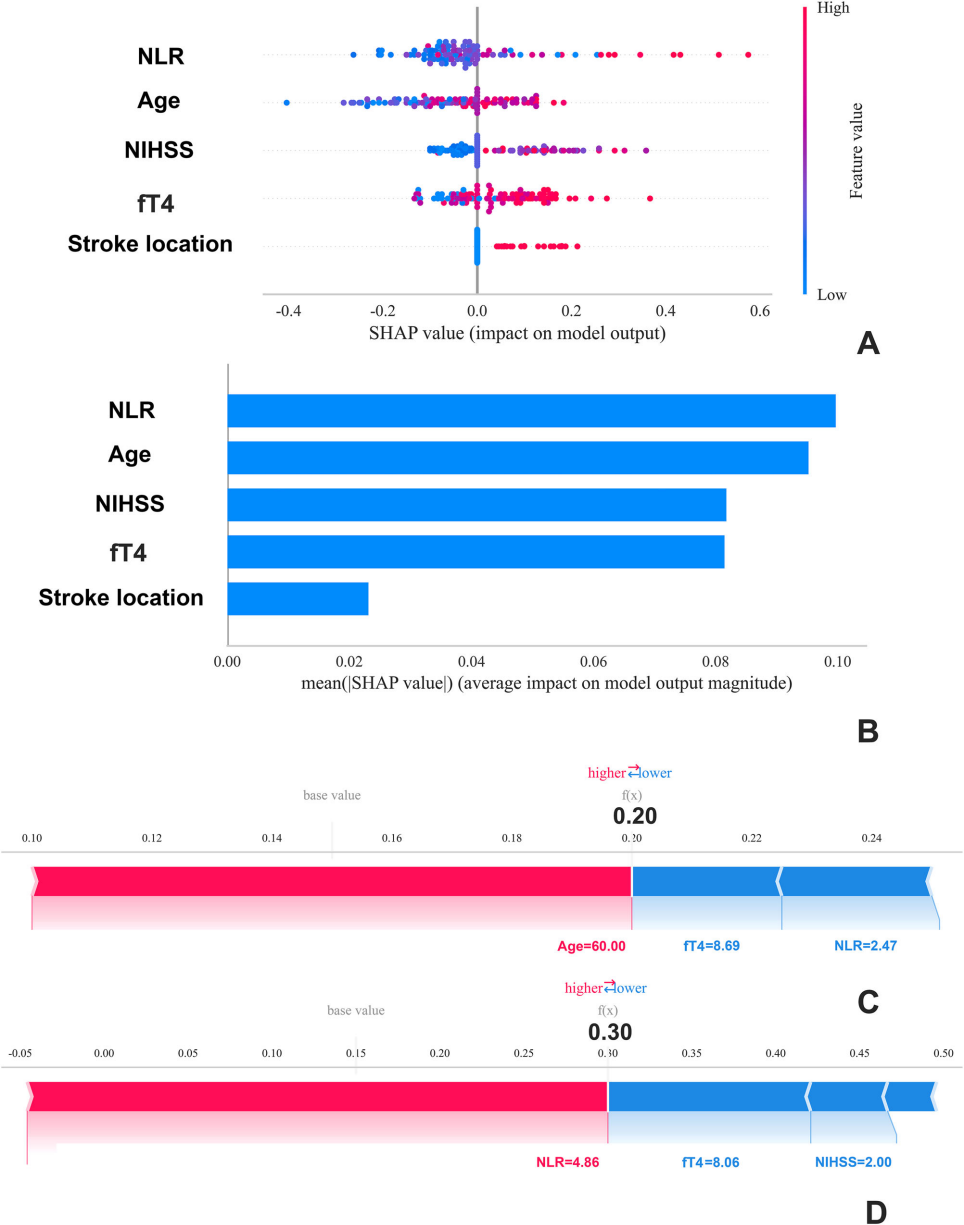
Summary plot of SHAP values. For each feature, one point corresponds to one patient. The position of point (A) on the x‐axis (i.e., the actual SHAP value) indicates the effect of the feature on the model output for that particular patient. Features are arranged along the y‐axis according to their importance, which is given by the average of their absolute Shapley values. The higher the position of the feature in the graph, the more important it is to the model (B). (C) and (D) show the SHAP force plot for two selected patients. Contributing variables are arranged on a horizontal line, ordered by the absolute value of their impact. The output value is the predicted NGT risk.

## 4 Discussion

Modern ML models, especially deep learning models and ensemble methods, are often characterized as “black boxes.” Despite their high predictive performance, their inherent complexity obfuscates the specific contribution of individual features to the prediction outcome. By quantifying the contribution of each feature to the model's prediction, SHAP helps us to open the “black box” and make the model more transparent, helping us to understand the model's decision‐making process. Increase the transparency and credibility of the model by explaining its predictions, which is especially important for explaining model decisions to nontechnical people.

We developed and validated a prognostic model using ML to predict swallowing function in patients with PSD and to guide NGT feeding decisions. Our findings indicate that approximately one‐fifth of AIS patients who fail to resume functional oral feeding 1 week post‐onset may benefit from NGT feeding intervention. Our model effectively predicts which patients will require an indwelling NGT at initial admission, demonstrating high discriminative ability with AUC values of 0.850 and 0.853 in the validation and test cohorts, respectively.

We acknowledge that the training‐set AUC of 1.000 indicates a degree of overfitting, which is an inherent characteristic of RF when applied to moderately sized datasets. The aggregation of numerous unpruned decision trees enables the model to memorize training patterns, including noise, resulting in the observed ∼15% performance gap between training and testing cohorts. However, the close agreement between fivefold cross‐validation performance (AUC = 0.850) and independent testing cohort performance (AUC = 0.853) provides reassurance that the model maintains acceptable generalizability. To mitigate overfitting, we applied hyperparameter constraints during model development, including limits on maximum tree depth and minimum samples per split and leaf. Nevertheless, some degree of overfitting persists. Future studies should explore stronger regularization strategies—such as gradient boosting with early stopping—and validate the model on larger, multicenter external datasets to further confirm its robustness.

The NIHSS score serves as a quantitative measure of neurological deficit severity and is widely utilized as an endpoint in clinical trials to stratify patients and inform therapeutic decision‐making. Similarly, when assessing swallowing function in patients after stroke, NIHSS appeared as a prognostic factor across studies (Jin et al. 2025; D'Netto et al. 2023; L. Wang et al. 2023). A significant limitation of the NIHSS score in dysphagia assessment is its suboptimal specificity, characterized by a negative predictive value below 40% despite a positive predictive value of approximately 90%. This diagnostic profile indicates that while patients exceeding the threshold score demonstrate high probability of dysphagia, those below the threshold cannot be reliably classified as non‐dysphagic. Consequently, the NIHSS should not be employed as a standalone screening parameter for dysphagia detection, but rather integrated as one component within a comprehensive assessment framework to facilitate evidence‐based clinical decision‐making. In this study, the NIHSS score was involved as part of a model to assess the likelihood of patients retaining NGT.

There is no conclusive evidence regarding the relationship between stroke location and swallowing function. Lesion sites varied across prediction models, including frontal cap, bilateral radial crown lesions (Galovic et al. 2019), basal ganglia or internal capsule (Lee et al. 2020), and cortex or brainstem (Z. Wang et al. 2022). Bilateral lesions have been consistently associated with poor swallowing recovery (Lee et al. 2020; Z. Wang et al. 2022). Oh et al. (2021) found that multifocal lesions were significant predictors of adverse swallowing outcomes. Contrary to these findings, our results did not find a worse outcome for multiple lesions, but rather correlated with stroke location. Our analysis revealed that AIS patients with infratentorial stroke demonstrated a 1.963‐fold higher probability of requiring prolonged NGT placement compared to those with supratentorial lesions. Studies have shown that lesions in the anterior and posterior central gyrus, the tecular area, the superior marginal gyrus, and the corresponding subcortical white matter tract of the right hemisphere are associated with PSD, and lesions in the posterior central gyrus are associated with severe dysphagia (P. Zhang et al. 2022; Lapa et al. 2021). The present investigation did not comprehensively examine these neuroanatomical correlations, highlighting the necessity for further detailed exploration in subsequent studies.

Age is the most studied demographic variable. One study reported youth as a positive predictor of recovery from dysphagia (Y. Li et al. 2023), while other studies found contrary to this (Z. Wang et al. 2022; Oh et al. 2021). Studies have reported that younger patients are more likely to have NGT removed before discharge than older patients. Functional independent scores for age and motor and cognitive ability predict oral feeding outcomes (Y. Li et al. 2023; Ikenaga et al. 2023). In the present investigation, we observed that advancing age was significantly associated with prolonged NGT dependency (OR: 1.074, 95% CI: 1.040–1.113), indicating better recovery patterns among younger patients. This is generally believed to be related to the faster recovery of younger patients from ischemic stroke.

In several previous studies, NLR was a stronger predictor of prognosis in patients with AIS than white blood cells, neutrophils, or lymphocytes alone. NLR reflects the balance between neutrophil and lymphocyte levels and their differential immune activity and has been used as a potential predictor of mortality in patients with AIS (Zuo et al. 2025; W. Zhang et al. 2020). Similarly, in this study, NLR was independently associated with NGT requirement after AIS (OR: 1.601, 95% CI: 1.343–1.942). As illustrated in Figure 4, the RF‐based prediction model identified NLR as the most influential predictor variable, demonstrating the highest feature importance score. However, we acknowledge that NLR likely serves as a marker of systemic pathophysiological alterations rather than a direct causative factor for dysphagia. AIS triggers a biphasic immune response: an early systemic inflammatory response syndrome (SIRS) characterized by neutrophil activation and release of pro‐inflammatory cytokines (IL‐6, TNF‐α, and IL‐1β), followed by a compensatory anti‐inflammatory response syndrome (CARS) manifested as lymphopenia and immunosuppression (Ascaso‐Vidal et al. 2026; Golenia and Olejnik 2026). Elevated neutrophil counts contribute to cerebral tissue injury through microvascular obstruction, blood‐brain barrier disruption, and amplification of neuroinflammation, potentially exacerbating neurological deficits including those affecting swallowing function (H. Wang et al. 2025). Concurrently, lymphopenia‐induced immunosuppression increases susceptibility to stroke‐associated pneumonia which occurs in up to one‐third of dysphagic stroke patients and can itself perpetuate or worsen swallowing dysfunction through prolonged inflammation, respiratory compromise, and reduced rehabilitation tolerance (Gao et al. 2025). Furthermore, NLR correlates strongly with stroke severity and infarct volume, both of which are established determinants of dysphagia (Olalusi et al. 2025). It is therefore plausible that the association between NLR and NGT requirement observed in our study is partially mediated through stroke severity rather than representing a direct causal pathway. In this context, NLR should be interpreted as a readily accessible composite biomarker that captures the combined influence of neuroinflammation, immunosuppression, and disease severity on poststroke functional outcomes.

Thyroid hormone levels have been shown to be associated with stroke outcomes in previous studies, but there are fewer studies specifically examining fT4. Some studies have shown that fT4 was significantly correlated with PSD, but its specific relationship with swallowing function has not been directly investigated, representing a gap that our findings begin to address. We caution that fT4 should not be interpreted as a direct cause of dysphagia. Rather, alterations in fT4 likely reflect dysregulation of the hypothalamic‐pituitary‐thyroid axis following acute stroke, a phenomenon well‐documented as non‐thyroidal illness syndrome or low‐T3 syndrome (Datta et al. 2023). AIS triggers profound neuroendocrine and metabolic stress responses, including altered peripheral conversion of T4 to T3 and changes in thyroid‐binding globulin levels (Datta et al. 2023). In this context, fT4 may serve as a surrogate marker for the magnitude of physiological stress and catabolic state following stroke, both of which are associated with larger infarct volumes and more severe neurological deficits. Beyond its role as a stress marker, thyroid hormones exert pleiotropic effects on the central nervous system, influencing neuroplasticity, axonal regeneration, cerebral glucose metabolism, and neurotransmitter synthesis. Altered thyroid hormone homeostasis may therefore impede neural repair mechanisms in brain regions governing swallowing coordination, including the brainstem swallowing center and cortical projection areas (Mele et al. 2025; X. Li et al. 2025). Additionally, thyroid dysfunction indirectly affects skeletal muscle metabolism, contractility, and fatigue resistance, properties critical for the oropharyngeal and esophageal phases of swallowing (Santa Maria and O'Dell 2024; Zhou et al. 2021). Cognitive impairment, which is more prevalent in patients with thyroid axis dysfunction, may also reduce cooperation with swallowing rehabilitation and oral feeding trials (X. Li et al. 2025; Zhou et al. 2021). Thus, the association between fT4 and NGT requirement in our model likely reflects the convergence of multiple indirect pathways rather than a direct causal relationship. Prospective mechanistic studies are warranted to disentangle these complex interactions.

This study has several limitations. First, it was a single‐center retrospective study of 553 patients, which may limit generalizability to other centers or populations and precludes causal inference. Unmeasured confounders and incomplete data recording are inherent risks of the retrospective design. Second, the training‐set AUC of 1.000 indicates overfitting, a recognized property of RF when applied to moderately sized datasets. Although hyperparameter constraints were applied, the gap between training and testing performance (∼15%) remains notable. More importantly, no external validation cohort was available; model robustness was assessed only via internal cross‐validation and a held‐out test set from the same center. Multicenter external validation is essential before clinical implementation. Third, FOIS assessment was integrated into routine clinical care without blinding, and formal inter‐rater reliability was not quantified. Patients who were lost to follow‐up (discharged, transferred, or deceased before 1 week) were excluded without imputation, which may have introduced selection bias.

## 5 Conclusion

We developed a model that predicts swallowing recovery and supports treatment decisions for AIS patients with swallowing disorders. The model individualizes treatment for each patient by identifying those who would benefit from enteral nutrition and retaining an NGT in place at the right time.

## Author Contributions

**Yaying Zeng**: conceptualization, methodology, validation, data curation, investigation, Writing – original draft. **Wenbo Zhang**: conceptualization, software, supervision, writing – review and editing, writing – original draft. **Yudi Han**: investigation, formal analysis, supervision, funding acquisition. **Yijia Ye**: investigation, project administration. **Peiliang Zhang**: data curation, supervision. **Wujie Shi**: methodology, project administration. **Jianbin Weng**: software, visualization. **Zhipeng Shen**: writing – review and editing, project administration, resources. **Zhi‐Lin Chen**: writing – review and editing, resources, funding acquisition, conceptualization, project administration, methodology.

## Funding

This work was supported by Hongkou District Health Committee (NO.2202‐37), the project of Shanghai Tongji Hospital (GJPY2425), the Project of Tongji University (2026‐0313‐YB‐11), and The Opening Foundation of State Key Laboratory of Neurology and Oncology Drug Development (SKLSIM‐F‐202432).

## Ethics Statement

The study was approved by the Ethics Committee of the Shanghai Tongji Hospital and complied with the Helsinki Declaration.

## Conflicts of Interest

The authors declare no conflicts of interest.

## Data Availability

The datasets used during the current study are available from the corresponding author on reasonable request.
